# Evaluation of Clinicopathological and Molecular Parameters on Disease Recurrence of Papillary Thyroid Cancer Patient: A Retrospective Observational Study

**DOI:** 10.3390/cancers12123637

**Published:** 2020-12-04

**Authors:** Salvatore Sorrenti, Giovanni Carbotta, Filippo Maria Di Matteo, Antonio Catania, Daniele Pironi, Francesco Tartaglia, Danilo Tarroni, Federica Gagliardi, Domenico Tripodi, Mikiko Watanabe, Stefania Mariani, Eleonora D’Armiento, Poupak Fallahi, Alessandro Sindoni, Corrado De Vito, Alessandro Antonelli, Salvatore Ulisse, Enke Baldini

**Affiliations:** 1 Department of Surgical Sciences, “Sapienza” University of Rome, 00161 Rome, Italy; salvatore.sorrenti@uniroma1.it (S.S.); giovanni.carbotta@uniroma1.it (G.C.); filippomaria.dimatteo@uniroma1.it (F.M.D.M.); Antonio.Catania@uniroma1.it (A.C.); daniele.pironi@uniroma1.it (D.P.); francesco.tartaglia@uniroma1.it (F.T.); danilo.tarroni@uniroma1.it (D.T.); federica.gagliardi@uniroma1.it (F.G.); domenico.tripodi@uniroma1.it (D.T.); enke.baldini@uniroma1.it (E.B.); 2 Department of Experimental Medicine, “Sapienza” University of Rome, 00161 Rome, Italy; mikiko.watanabe@uniroma1.it (M.W.); s.mariani@uniroma1.it (S.M.); 3 Department of Internal Medicine and Medical Specialties, “Sapienza” University of Rome, 00161 Rome, Italy; eleonora.darmiento@uniroma1.it; 4 Department of Clinical and Experimental Medicine, University of Pisa, 56126 Pisa, Italy; poupak.fallahi@unipi.it (P.F.); alessandro.antonelli@med.unipi.it (A.A.); 5 Department of Public Health and Infectious Diseases, “Sapienza” University of Rome, 00161 Rome, Italy; alessandro.sindoni@uniroma1.it (A.S.); corrado.devito@uniroma1.it (C.D.V.)

**Keywords:** papillary thyroid cancer, prognosis, TNM, histology, multifocality, lymph node metastasis, vascular invasion, autoimmune thyroid diseases

## Abstract

**Simple Summary:**

Papillary thyroid cancer (PTC) patients are staged according to the Tumor-Node-Metastasis staging system (TNM). This work was aimed at comparing the usefulness of the 8th edition of TNM (TNM-8), currently used, and that of the previous one (TNM-7) for predicting disease-free interval (DFI) in a cohort of 1148 patients. Moreover, clinicopathological and molecular factors were statistically evaluated in order to determine which of these was/were the best predictor(s) of DFI. Results obtained from the multivariate analysis indicated that advanced tumor stages were independent risk factors for a lower DFI regardless of TNM, but the statistical model created with the TNM-7 was most accurate. When stage-determining factors were included individually in the multivariate analysis, LN metastases, tall cell variant, and age emerged as independent risk factors for a shorter DFI, with lateral LN metastases being the most relevant. No molecular parameters could improve the prediction of DFI provided by LN metastases.

**Abstract:**

The American Joint Committee on Cancer has revised the Tumor-Node-Metastasis (TNM) staging system for papillary thyroid cancer (PTC) patients. We examined the impact of this new classification (TNM-8) on patient stratification and estimated the prognostic value of clinicopathological features for the disease-free interval (DFI) in a cohort of 1148 PTC patients. Kaplan–Meier analyses showed that all clinicopathological parameters analyzed, except age and multifocality, were associated significantly with DFI. Cox regression identified tall cell PTC variant and stage as independent risk factors for DFI. When the stage was replaced with age, tumor size, and lymph node (LN) metastases in the set of covariates, the lateral LN metastases stood out as the strongest independent predictor of DFI, followed by tall cell variant and age. A noteworthy result emerging from these analyzes is that regression models had lower Akaike and Bayesian information criterions if variables were categorized based on the TNM-7. In addition, we examined data from a different PTC patient cohort, acquired from The Cancer Genome Atlas database, to verify whether the DFI prediction could be enhanced by further clinicopathological and molecular parameters. However, none of these was found to be a significant predictor of DFI in the Cox model.

## 1. Introduction

Accurate staging of patients affected by differentiated thyroid cancer (DTC) is of crucial importance to ensure the appropriate therapeutic strategy and follow-up, and to ensure the patients’ quality of life [1]. To date, several staging systems aimed at estimating the risk of disease-related death or disease relapse/persistence are available. The most employed one is the Tumor-Node-Metastasis (TNM) classification, developed by the American Joint Committee on Cancer (AJCC) [2,3]. The latest version of the TNM system (8th edition/TNM-8) was significantly amended in 2016 with respect to the previous version (7th edition/TNM-7) released in 2009 [3,4]. The 8th edition, while maintaining the classical anatomic extension of the disease as its groundwork, incorporates biological and molecular markers to create a more personalized prognostic stratification [4]. Based on TNM-8, many DTC patients are now included in lower stages and considered to have a reduced risk of dying from thyroid cancer [3,5]. However, in DTC patients the risk of disease recurrence is considerably higher than the risk of disease-related mortality, which makes the TNM staging system, designed to foresee patient survival, uninformative for the prediction of disease recurrence [1,6]. In 2009 the American Thyroid Association (ATA) endorsed a validated risk-stratification system for DTC recurrence in which TNM parameters were implemented by clinicopathological features (i.e., tumor histology, vascular invasion, radioactive iodine uptake, post-operative thyroglobulin serum level, etc.) to divide patients in three risk categories (low, intermediate, and high) [6,7,8]. Although this model was recognized as a valuable tool in clinical practice, in 2015 the ATA substituted the three-risk-categories-model with a continuum risk model varying from very low risk to high risk of recurrence [1,5]. In the latter, besides TNM and clinicopathological parameters, mutations of BRAF and TERT genes were included [1,6]. In the TNM-8 it has been recommended to take note, in individual patient records, of a number of molecular and clinicopathological parameters that, even if not included in the actual staging system, could be evaluated for inclusion in the next TNM edition [3,4]. They comprise the microscopic extrathyroidal extension, location and number of metastatic lymph nodes, number of lymph nodes sampled and size of the largest metastatic one, extranodal extension, histological subtypes, vascular invasion, postoperative thyroglobulin (Tg) serum level, extension of surgical resection, and molecular characterization [3,9]. In the present work, we retrospectively investigated a case study comprising 1148 patients affected by papillary thyroid cancer (PTC) to evaluate: (i) the effect of the new TNM-8 staging system on patients’ risk stratification compared to the previous TNM-7; (ii) the prognostic value of a number of clinicopathological and molecular parameters determined by proportional hazards regression (Cox regression).

## 2. Results

**Figure 1:**
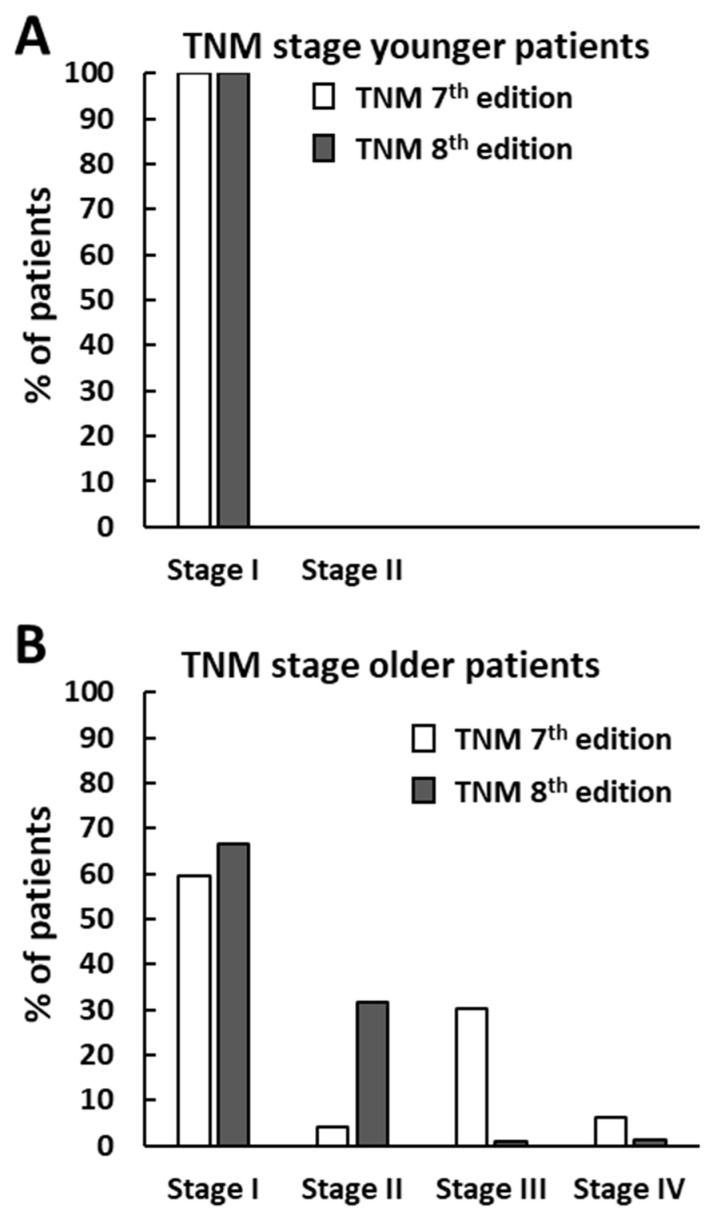
Distribution of 1113 papillary thyroid cancer (PTC) patients according to the 7th or the 8th Tumor-Node-Metastasis (TNM) staging system. (**A**) displays the staging of patients younger than 45 years (516 out of 1113 patients) according to the TNM-7 or younger than 55 years (764 out of 1113 patients) according to the TNM-8. (**B**) displays the distribution of stages for older patients as defined in the 7th or the 8th TNM edition.

We first evaluated the impact of the new TNM-8 on patients’ risk stratification compared to the previous TNM-7. In this regard, 1113 patients for whom all clinical data required were available, were staged according to both TNM systems. Since in the TNM-8 the patients’ age cutoff has been shifted from 45 to 55 years, the percentage of younger patients (age < 55 years) in our case study increased from 46.4% (516/1113) in TNM-7 to 68.6% (764/1113) in TNM-8. Due to the lack of distant metastases, all these patients were classified as stage I regardless of TNM edition. As expected, among older patients (age > 55 years) we observed a considerable reduction in the relative frequencies of individuals at stage III and IV, with a concomitant increase in those at stage I and II, moving from TNM-7 to TNM-8 (Figure 1). As noted in Figure 1, classification of PTC according to TNM-8 led to a considerable downstaging of patients.

Univariate analysis was performed to evaluate the association of several clinicopathological parameters, including age at diagnosis, gender, autoimmune thyroid disease (AITD), tumor histology, size (T), lymph node metastases (N), stage, multifocality, capsular, muscle, and vascular invasion, with PTC recurrences. Since some categories were poorly represented, especially among tumor sizes and stages, they were combined with other categories to avoid the inclusion of too small groups in the statistics (see Section 4). As shown in Table 1, all the parameters analyzed with the exclusion of age and multifocality were significantly associated with PTC recurrences. In particular, the PTC sclerosing and tall cell variants and lymph node metastases had a very strong correlation with recurrences (Cramer’s V index > 0.25). 

**Figure 2:**
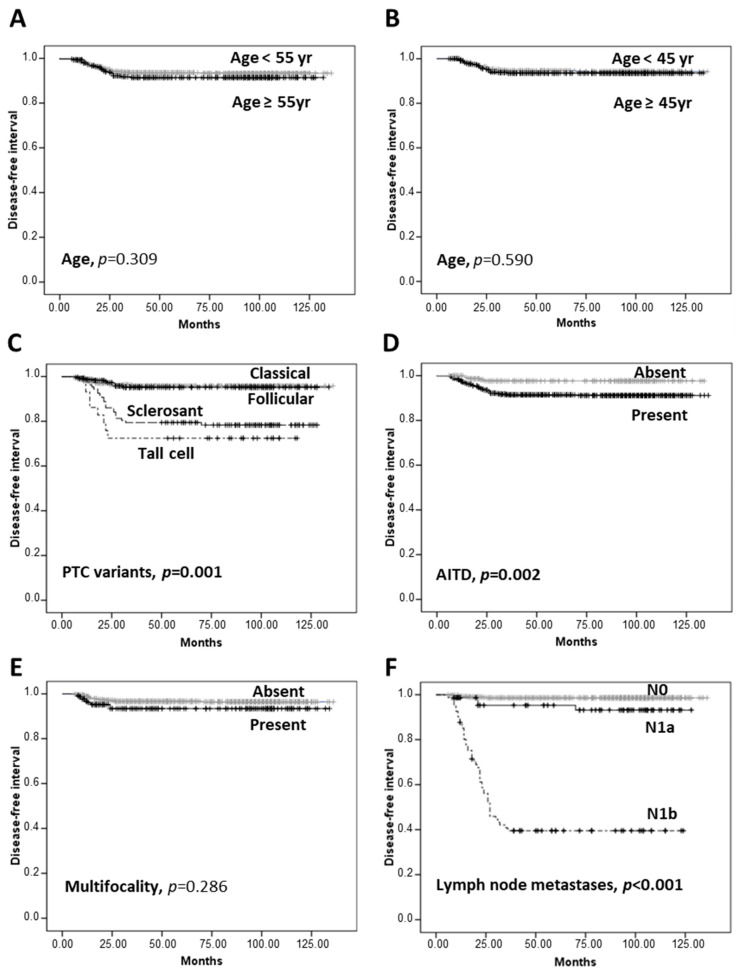

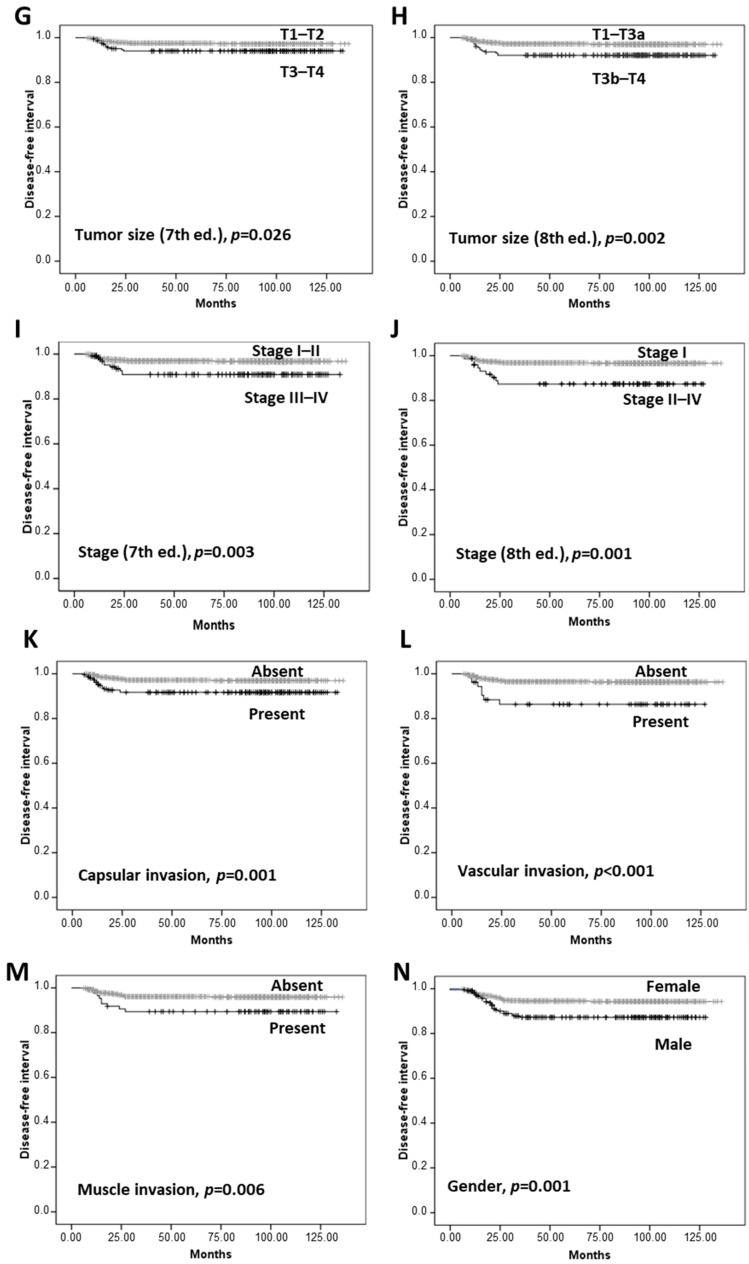
Kaplan–Meier analysis. Kaplan–Meier curves with Mantel–Cox log-rank statistical text were made to estimate the impact on disease-free interval (DFI) at age <55 years or ≥55 year (**A**), age <45 years or ≥45 years (**B**), PTC histological variants (**C**), AITD (**D**), multifocality (**E**), lymph node metastases (**F**), tumor size (7th edition) (**G**), tumor size (8th edition) (**H**), stage (7th edition) (**I**), stage (8th edition) (**J**), capsular invasion (**K**), vascular invasion (**L**), muscle invasion (**M**), or gender (**N**). *p* values < 0.05 are evidenced in bold.

These observations were confirmed by the Kaplan–Meier analysis, reported in Figure 2. All clinicopathological parameters, with the exception of age and multifocality, were found to impact significantly on disease-free interval (DFI). Patients classified as T1a and T1b had the same DFI. 

We finally created Cox regression models to predict the probability of DFI as a function of different sets of independent variables. Clinical parameters were categorized as in the univariate analysis (see Table 1). The first series of covariates included gender, histological variants (classical, follicular, sclerosing, and tall cell), AITD, multifocality, stage, capsular, muscle, and vascular invasion. Tall cell variant and stage turned out to be significant predictors of DFI (see Table 2), and the stage was significant either if calculated with the 7th or with the 8th edition. Of note is that using TNM-7 instead of TNM-8, the Akaike information criterion (AIC) decreased by about 12 units, indicating a significant improvement of the model.

We then replaced stage with stage-related parameters, so that the new set of covariates comprised lymph node metastases, tumor size, dichotomized age at diagnosis, gender, histological variants, AITD, multifocality, muscle and vascular invasion (see Table 3). Capsular invasion was excluded due to its collinearity with tumor size (variance inflation factor > 5). In this setting, the presence of lymph node metastases beyond the central compartment (N1b) was the independent variable most strongly associated with DFI (Hazard Ratio = 37.55, *p* < 0.001). Although in the univariate analysis dichotomous age was not significantly associated with recurrences or DFI, in Cox regression it became a significant predictor both applying the 55-year and 45-year threshold. When the hazard function was generated with T and age categorized according to the TNM-7 edition, the sclerosing and tall cell variants also displayed significant hazard ratios. Moreover, this model had lower AIC and BIC than that resulting from categories based on TNM-8, similarly to what was observed for the Cox regression including stage.

After that, we sought to verify if the DFI prediction could be strengthened by combining lymph node metastases and age with further histological and molecular parameters that were not available for our patients. These data were acquired from a previous study by the Cancer Genome Atlas Research Network for Cancer Genomics [10,11,12]. Specifically, we considered lymph node metastases, age, number of total non-silent mutations, number of CpGT mutations, BRAF-RAF score, ERK score, miRNA cluster, RPPA cluster, ploidy, differentiation score, and follicular component. Univariate analysis showed that BRAF-RAF score (*p* = 0.014), differentiation score (*p* = 0.045), and lymph node metastases (*p* = 0.005) associated significantly with recurrences. Nonetheless, adding all the above variables with *p* < 0.25 in the univariate analysis to lymph node metastases and age in the Cox regression did not improve significantly the prediction of DFI. Considering a subgroup of patients (*n* = 310) for which lymph node metastases were categorized as N0, N1a, and N1b in the database, N1b displayed the highest hazard ratio and significance, similar to what emerged from the analysis of our patient cohort (Table 3). In particular, N1a showed a HR of 6.53 (95% CI 1.14–37.37, *p* < 0.05) and N1b had a HR of 14.46 (95% CI 2.29–91.07, *p* < 0.01).

## 3. Discussion

Follicular thyroid cell-derived tumors are the most common endocrine malignancy and the fifth most common cancer in women [13,14,15]. Its annual incidence, about 3% of all cancer, has been shown to more than double over the last decades as a result of the improved ability to diagnose malignant transformation in small thyroid nodules [14,15]. The differentiated papillary (PTC) and follicular (FTC) carcinomas represent most of the epithelial thyroid cancers, which may progressively dedifferentiate giving rise to the more aggressive poorly DTC (PDTC), and to the incurable anaplastic thyroid carcinomas (ATC) [16]. Although the prognosis of DTC patients is favorable, with a 5-years-survival rate of nearly 98%, about 20% of them face the morbidity of disease recurrence [1,2,14,15]. The TNM staging system elaborated by the AJCC based on clinicopathological parameters is the most widely used approach to predict thyroid cancer survival, but it is much less reliable in discriminating patients with a higher risk of developing relapses over time [1,2,3]. The TNM has been significantly revised in 2016 (TNM-8), with respect to its previous version (TNM-7) [3,4,9]. Various reports have documented the ability of the TNM-8 to better predict the disease-specific survival (DSS) in DTC patients, but we could not verify this point because none of the patients examined died due to PTC [17,18,19,20,21,22,23,24,25,26,27,28]. It has to be mentioned, however, that in one of these studies it was shown that the new TNM improved the prediction of DSS for PTC, but not for FTC patients [23]. In addition, reasonable concerns have been raised for patients in the 45–54 age range, classified in stages III or IV by the TNM-7 but currently considered in stages I or II by the TNM-8, for whom the severity of the disease could be underestimated [24].

In the present study, as expected, the comparison of the two TNM systems in 1113 PTC patients evidenced a considerable patient downstaging by TNM-8, in agreement with a number of earlier reports [5,17,18,19,20,21,22,23,24,25,26,27,28]. Even though TNM staging was conceived to predict DSS, some studies showed that both TNM-7 and TNM-8 were significantly associated with disease-free survival and that TNM-8 allowed better discrimination of the recurrence risk over time [25,27,29]. In our case study we found analogous results, but the differences between curves created with TNM-7 and TNM-8 stages and T were much less evident in our Kaplan–Meier analysis due to the need for combining some categories to fulfill statistical requirements. However, when the Cox regression analysis was performed with parameters classified according to TNM-7 instead of TNM-8, lower Akaike Information Criterion (AIC) and Bayesian Information Criterion (BIC) values were obtained, indicating that the model based on TNM-7 should be preferred. This statement would seem to be in contrast with the conclusions of other authors about the superior predictive performance of TNM-8 over TNM-7 for DFI. However, it has to be mentioned that these authors focused attention on HRs of individual predictors, while we evaluated the virtue of model fit. Our findings are in line with a previous work showing that in patients above 55 years, pT7 was superior to pT8 in predicting DFI [28].

In the framework of The Cancer Genome Atlas (TCGA) project, a comprehensive multiplatform analysis was carried out to determine the genomic landscape of 496 PTC cases, and a reclassification of PTC into molecular subtypes was proposed to improve clinicopathological grading and management of patients [12]. In this study, the lowest thyroid differentiation score (TDS) was assigned to a tall cell-like tumor cluster, which was associated with more advanced stage and higher risk, while the classical PTC (CPTC) had an intermediate TDS, and the follicular variant (FVPTC) maintained a high TDS [12]. These results were corroborated by a subsequent multicenter retrospective study including 6282 cases of PTC [29]. Differential risk patterns of disease recurrence and patient mortality were determined for the three major PTC variants, with increasing aggressiveness from the FVPTC to the CPTC, up to the tall cell PTC (TCPTC) [30]. The results of our study do not confirm a higher recurrence-free probability for FVPTC compared to CPTC, whereas the DFI probability was significantly reduced for the tall cell and sclerosing PTC variants. The partial discrepancy of our findings with the previous ones is probably because, due to the numerical scarcity of relapses in FVPTC, we could not distinguish between encapsulated/well-demarcated noninvasive forms and infiltrative FVPTC.

When in our regression analysis the TNM stage was replaced by stage-related parameters analyzed as independent variables, lymph node metastasis and age dichotomized in <55 years and ≥55 years emerged as the only significant predictors of DFI. Taking advantage of a PTC cohort of patients derived from the TCGA, we sought to verify whether, besides lymph node metastasis and age, the DFI prediction could be improved by additional histological and molecular parameters not available for our case study. The results obtained demonstrated that only a couple of them (BRAF-RAF score and differentiation score) were significantly associated with recurrences, and none of the parameters selected for the Cox regression impacted significantly on the model. Additionally, in this PTC cohort, lateral lymph node involvement represented the best predictor of DFI, with the N1b category (spreading beyond the central compartment) having the highest HR.

## 4. Materials and Methods

### 4.1. Patients

In this study, we retrospectively analyzed 1148 patients affected by PTC enrolled in the period 1995–2018 at the Umberto I hospital, Department of Surgical Sciences of the Sapienza University of Rome. The case study included 883 females and 265 males with a median age of 47 years (range 12–88 years). All subjects gave their informed consent for inclusion before they participated in the study. The study was conducted in accordance with the Declaration of Helsinki, and the protocol was approved by the Ethics Committee of the Umberto I hospital (Protocol No. 2615). Of the 1148 patients, 1127 patients had a total thyroidectomy, while 21 patients had a lobectomy. Post-surgical RAI was performed on 565 patients, but not on 583 patients including 486 pT1a cases and 97 pT1b cases. Thyroid hormone replacement was applied following international guidelines recommendations [1,2,3,31,32]. For each patient, data regarding age at diagnosis, gender, absence or presence of autoimmune thyroid disease (AITD), tumor size, occurrence of lymph nodal or distant metastases, histology, multifocality, capsular invasion, vascular invasion, and muscle infiltration were collected. All patients were staged according to either the TNM-7 or the TNM-8. Histopathological diagnoses were made based on the WHO classification [11]. In particular, the main PTC variants observed included: 683 classical, 279 follicular, 121 diffuse sclerosing, and 32 tall cell variants. Of the 1148 patients, 283 were affected by AITD, 276 by chronic lymphocytic thyroiditis, and 7 by Graves’ disease. The follow-up was available for 964 patients with a mean duration of 69.51 months (range 12–136 months, Table 1). Of these, 13 patients died for causes not related to PTC. In patients not affected by AITD, recurrences were diagnosed by neck ultrasonography, measurement of serum Tg levels, either in basal conditions or following recombinant human TSH stimulation (rhTSH), fine needle aspiration cytology (FNAC), and/or Tg determination in the FNA wash-out from lymph nodes [33], ^131^I whole-body scan, or histological analysis following surgical resection of the lesion. The same was applied to AITD patients with positive Tg auto-antibodies with the exclusion of Tg measurements. During the follow-up, we observed 11 persistence (diagnosed before 12 months from the initial therapy) and 55 recurrences, including 54 lymph node metastases and 1 lung metastasis. In addition, we used data previously obtained from a study that described the genomic landscape of 496 PTC, with 396 of which follow-up was available [11,12,13,14].

### 4.2. Statistical Analysis

To avoid bias in the univariate and multivariate analyses, variable categories containing a small number of events, whose statistical results were unreliable, were merged with larger categories. Thus, in accordance with TNM-7, we divided tumor sizes (T) in T1 + T2 and T3 + T4, and tumor stages (S) in S1 + S2 and S3 + S4. The tumor sizes as defined by the TNM-8 were classified in T1 + T2 + T3a and T3b + T4, and the tumor stages in S1 and S2 + S3 + S4. Univariate analyses were executed for each clinical parameter in relation to the presence/absence of recurrence. Statistical comparison between recurrent and non-recurrent patients was carried out with the Chi-square test or the Fisher exact test for categorical variables, and with the Mann–Whitney test for continuous variables (not normally distributed). Where a significance was found in the Chi-square test, the Cramer’s V was used as a post-test to determine the strength of association between variables. Kaplan–Meier curves were created to estimate the DFI in patient groups, and differences were statistically evaluated by the log-rank test. Finally, Cox regression was performed to quantify the hazard ratios of several explanatory variables, both continuous and categorical. The selection of covariates was made by including all parameters with a *p*-value < 0.25 in the univariate analysis, together with those of known clinical significance. Proportional hazards assumption and absence of multi-collinearity were preliminarily assessed for these covariates. The backward stepwise approach was used for the model selection. Data analyses were done by using the SPSS software for Windows (SPSS, Inc., Chicago, IL, USA), considering the probability value <0.05 as the threshold limit for statistical significance.

## 5. Conclusions

In conclusion, our study evidenced that lymph node metastases display a predictive value for DFI of PTC patients far higher than other clinical, histological, and molecular parameters currently available. Hence, it would be desirable to try to further improve the prediction of the DFI by employing more in-depth analysis of lymph node characteristics, through both instrumental and molecular investigations. 

## Figures and Tables

**Table 1:** Univariate analysis of the association between clinicopathological parameters and PTC recurrences. AITD, Autoimmune Thyroid Disease. *p*-values < 0.05 are evidenced in bold.

Clinicopathological Parameters	No Recurrences	Recurrences	Cramer’s V Index	*p*-Value
Median age (range)	47 year (12–80 year)	48 (23–85 year)	-	0.906
**Age**				
≥55 year	621	32	-	0.118
<55 year	288	23		
≥45 year	419	25	-	0.926
<45 year	490	30		
**Gender**				
Male	192	24	0.125	**<0.001**
Female	717	31		
**AITD**				
Yes	238	5	0.091	**0.005**
No	671	50		
**PTC variants**				
classical	545	16	0.291	**<0.001**
follicular	232	8		
sclerosing	84	23		
tall cell	21	7		
**T** (7th edition)				
T1–T2	716	12	-	**0.028**
T3–T4	193	9		
**T** (8th edition)				
T1a–T3a	732	15	0.094	**0.004**
T3b–T4	177	11		
**N**				
N0	663	7	0.647	**<0.001**
N1a	71	3		
N1b	33	42		
**Stage** (7th edition)				
Stages I–II	787	15	-	**0.001**
Stages III–IV	120	10		
**Stage** (8th edition)				
Stage I	844	18	-	**<0.001**
Stages II–IV	65	8		
**Multifocality**				
Yes	208	8	-	0.412
No	701	19		
**Capsular invasion**				
Yes	197	11	0.077	**0.019**
No	712	16		
**Muscle invasion**				
Yes	76	7	-	**0.015**
No	833	24		
**Vascular invasion**				
Yes	46	5	-	**0.013**
No	863	22		

**Table 2:** Cox regression for the prediction of DFI was performed with stage and other clinicopathological parameters. The table shows predictors retained in the model after backward selection. The variables entered in the analysis were: gender, histological variants, AITD, multifocality, stage (8th or 7th edition), capsular, muscle, and vascular invasion. HR: hazard ratio. CI: confidence interval. AIC: Akaike information criterion. BIC: Bayesian information criterion. *p*-values < 0.05 are evidenced in bold.

**Cox Regression with TMN-8**					
**Clinicopathological Parameters**	**HR**	**95% CI**	***p*-Value**	**AIC**	**BIC**
Sex	2.207	0.96–5.06	0.062	308.74	343.1
Histological variant					
Classic	1	---	---		
Follicular	0.57	0.16–2.03	0.387		
Sclerosing	2.22	0.81–6.08	0.122		
Tall-cell	4.08	1.12–15.10	**0.035**		
Stage	4.28	1.76–10.43	**0.001**		
**Cox Regression with TMN-7**					
**Clinicopathological Parameters**	**HR**	**95% CI**	***p*-Value**	**AIC**	**BIC**
Histological variant				296.403	315.89
Classic	1	---	---		
Follicular	0.66	0.18–2.37	0.524		
Sclerosing	2.43	0.88–6.69	0.085		
Tall-cell	4.31	1.17–15.83	**0.028**		
Stage	1.95	1.27–2.98	**0.002**		

**Table 3:** Cox regression for the prediction of DFI performed with stage-determining factors and other clinicopathological parameters. The table shows predictors retained in the model after backward selection. The variables entered in the model were: gender, histological variants, AITD, multifocality, dichotomized age, T, N, muscle and vascular invasion. HR: hazard ratio. CI: confidence interval. AIC: Akaike information criterion. BIC: Bayesian information criterion. *p*-values < 0.05 are evidenced in bold.

**Cox Regression with TMN-8**					
**Clinicopathological Parameters**	**HR**	**95% CI**	***p*-Value**	**AIC**	**BIC**
Age (<55 year and ≥55 year)	2.907	1.24–6.81	**0.014**	238.59	253.2
Lymph node metastasis					
N0	1	---	---		
N1a	3.75	0.77–18.24	0.101		
N1b	37.55	14.79–95.34	**<0.001**		
**Cox Regression with TMN-7**					
**Clinicopathological Parameters**	**HR**	**95% CI**	***p*-Value**	**AIC**	**BIC**
Age (<45 year and ≥45 year)	3.5	1.16–10.55	**0.026**		
Histological variant				202.97	241.94
lassic	1	---	---		
Follicular	1.53	0.37–6.29	0.556		
Sclerosing	3.42	1.04–6.69	**0.042**		
Tall-cell	7.22	1.69–30.80	**0.008**		
T (T1–T2 and T3–T4)	0.607	0.343–1.075	0.087		
Lymph node metastasis					
N0	1	---	---		
N1a	4.22	0.84–21.27	0.081		
N1b	34.72	10.43–115.601	**<0.001**		
